# Supplementary material for: Preparation of Superhydrophobic Materials and Establishment of Anticorrosive Coatings on the Tinplate Substrate by Alkylation of Graphene Oxide
Source: Polymers (Basel). 2023 Mar 3;15(5):1280. doi: 10.3390/polym15051280 (PMC10007501; doi:10.3390/polym15051280)
Supplement: Supplementary file 1 [file polymers-15-01280-s001.zip › polymers-2151021-supplementary.pdf]

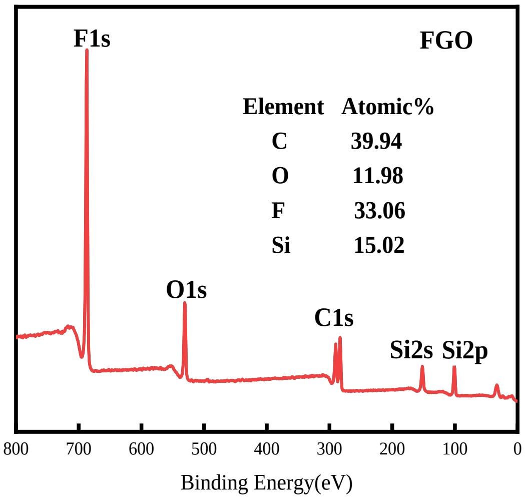

Figure S1. XPS spectrum of FGO

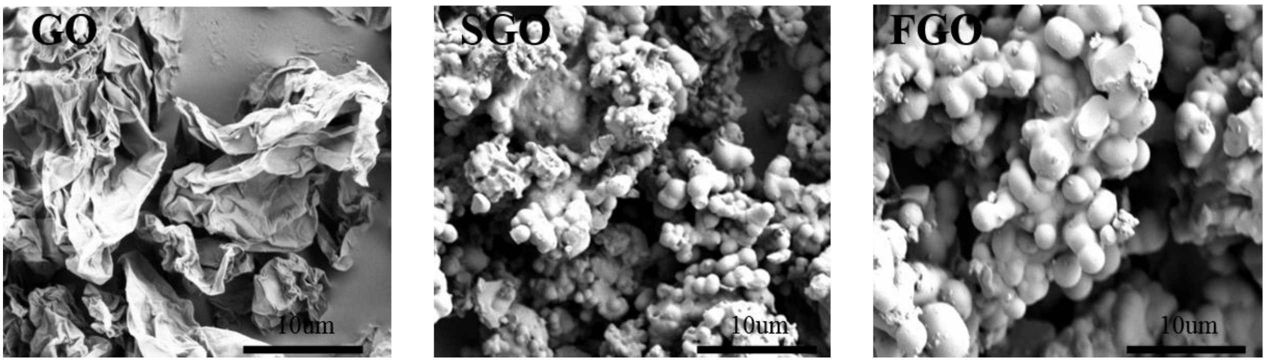

Figure S2. SEM of GO, SGO, FGO.

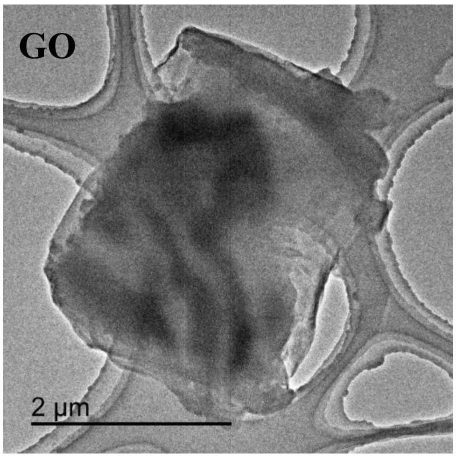

Figure S3. TEM of GO.

Table S1. Effect of water droplet residence time on FGO coating on water contact angle and rolling angle

T(d)	CA (°)	SA (°)
1	151.3°	3.9°
3	150.9°	4.2°
5	150.1°	4.8°
10	144.7°	5.3°

Note: Time in days (T), superhydrophobic water contact angle (CA), superhydrophobic water rolling angle (SA).

Table S2. This work and related literature hydrophobic properties and corrosion resistance comparison table

Sample	CA	$N_{\text{relative } I_{corr}}$	Reference
ATGO	_	2	[22]
GLGO	_	1	[23]
Silane/GO	< 151°	-	[25]
SF-GO	_	4	[39]
FGO	151.3°	3	Our work

The difference between the current density of the composite coating and the current density of the unmodified epoxy coating （Nrelative Icorr), Superhydrophobic water contact angle (CA), The non-superhydrophobic and unmeasured composite coatings (—).
